# Food Packaging and Chemical Migration: A Food Safety Perspective

**DOI:** 10.1111/1750-3841.70265

**Published:** 2025-05-22

**Authors:** Nurbanu Seref, Gizem Cufaoglu

**Affiliations:** ^1^ Department of Veterinary Food Hygiene and Technology, Institute of Health Sciences Kırıkkale University Kırıkkale Turkey; ^2^ Department of Food Hygiene and Technology, Faculty of Veterinary Medicine Kırıkkale University Kırıkkale Turkey

**Keywords:** food preservation, migrant, packaging‐food interactions, public health, toxic substances

## Abstract

Packaging has become an essential component of food production and distribution. It plays a vital role in preserving food quality and safety, while also helping to reduce food waste. However, the widespread use of packaging has led to increased chemical migration, posing significant risks to food safety and public health. Migration occurs when low molecular weight compounds from packaging materials, printing inks, or adhesives transfer to food under certain conditions, potentially introducing harmful substances. This contamination can degrade food quality through unwanted changes and expose consumers to serious health risks, highlighting the need for stringent controls. This review examines various packaging materials, explores the factors influencing the migration of chemical substances from packaging into food, compiles data on the presence of migrants in foods, identifies those posing risks to public health, and underlines measures to minimize migration from a food safety perspective.

## 1 Introduction

Food, one of the most basic human needs, has become almost inseparable from packaging. The need for packaging has existed for thousands of years, and people have historically relied on a variety of methods to meet this requirement, including dried pumpkins, coconut shells, leaf pots, and pottery (Bansal and Gupta 2020). Today, population growth, fast‐paced lifestyles, advances in plastic recycling infrastructure, and ease of transportation have significantly increased the demand for packaged foods and takeaway services. As a result, packaged foods now make up a significant portion of the food consumed.

Although the primary function of packaging seems to be to preserve food, it also plays an important role in preserving the taste, nutritional value, and freshness of food. By extending shelf life and maintaining quality, packaging contributes to reducing food. However, in addition to these advantages, the possibility of the components contained in the packaging passing into the food reveals the risk of contamination, which causes changes in the organoleptic properties, quality, and safety of the food, as well as the exposure of people to chemicals (Karakoç and Dikmen 2024). The passage of chemical compounds in the packaging material into food under certain conditions is called migration (Muncke et al. 2020). These compounds have a low molecular weight and are usually caused by packaging materials, printing chemicals, and adhesives (Yenidoğan et al. 2023). Materials that come into contact with food should be free of components that may pose a health risk and should not cause adverse changes in the food. The migration of harmful ingredients affects food safety, whereas the migration of substances such as stains or odorants affects food quality. These problems may arise during the production, transportation, storage, preparation, or consumption stages of food. Uncontrolled contact between packaging and food can lead to rapid increase in contamination and eventually reach legal limits (Yenidoğan et al. 2023).

Substances that migrate to the human body through food can exhibit hazardous properties such as bioaccumulation, persistence, carcinogenicity, endocrine disruption, and mutagenicity. These pose significant risks to human health and may contribute to the development of non‐communicable chronic diseases (Karakoç and Dikmen 2024; Geueke et al. 2024). Moreover, even if these amounts do not pose immediate health risks, migration can lead to economic losses. For instance, in 2010, approximately 28 million boxes of grain‐based food products were recalled due to excessive amounts of methylnaphthalene leaching from packaging (Snedeker 2014).

Factors such as the functional properties of the packaging material, the intrinsic properties of the food (e.g., pH, water activity), external factors (e.g., temperature, atmosphere), storage conditions, and shelf life play a critical role in the selection of appropriate packaging materials. Although a wide variety of materials are used in direct contact with food, the most common ones are plastic, glass, and paper (EPA 2020). These materials offer their own advantages and disadvantages. For example, glass retains the flavor of food without changing its quality. Paper and cardboard are lightweight and printable materials that can be produced at low cost and also provide practicality in terms of transportation. Metal like iron and aluminum are values for their recyclability, sealing properties, and malleability. Additionally, aluminum can easily adhere to paper or plastic surfaces, enabling the creation of diverse packaging designs. Plastic, on the other hand, has become the dominant material in the food packaging industry due to its durability, low cost, lightweight, and easy formability, making it the primary choice for packaging (Bansal and Gupta 2020). Furthermore, various substances such as printing inks, varnishes, and waxes can also come into contact with food and are prone to migrate (Nerin et al. 2013). In this review, it was aimed to explore packaging materials, examine the factors influencing chemical migration into food, compile data on the presence of harmful migrants in foods, and underline the measures to minimize migration to ensure food safety.

## 2 Factors Affecting Migration

**FIGURE 1:**
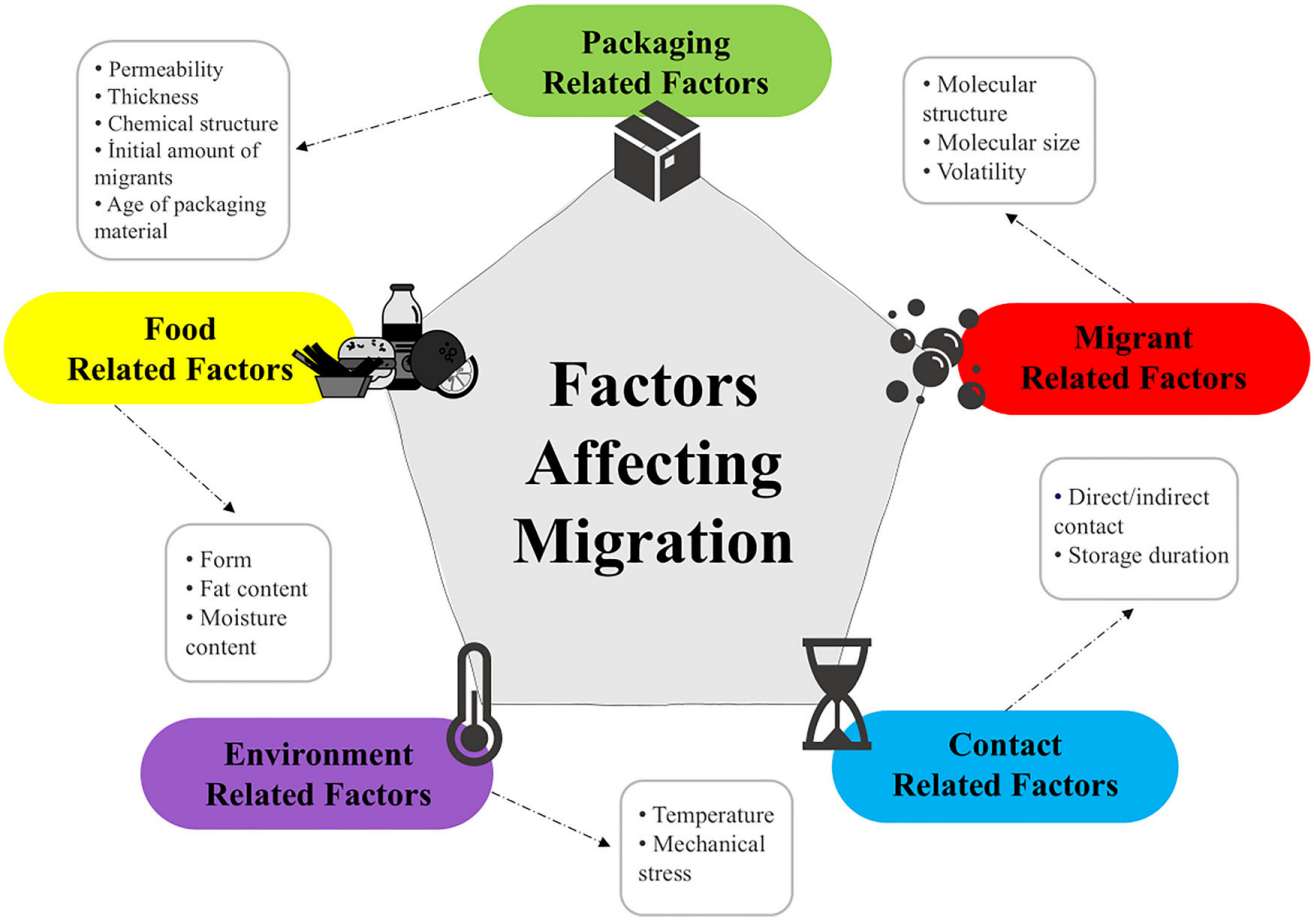
Factors affecting migration.

Migration is the absorption of chemicals in packaging material by food (or vice versa) through a specific process (Alamri et al. 2021). Migration from food packaging material refers to the transfer of chemical substances in the packaging material to food and is a critical issue in terms of food safety. Factors affecting migration include temperature, contact time, chemical composition of the packaging material, structure of the food (e.g., fat content, water activity, acidity), and the size of the contact surface between the packaging and the food (Kato and Conte‐Junior 2021). These factors are shown in Figure 1.

Factors such as form of the food, fat and moisture content, and surface area can significantly influence migration (BRCGS 2017). For example, the fat content in food can enhance the mobility of lipophilic substances within the packaging by facilitating their migration, as the transfer of fat from the food to the packaging material promotes their movement back into the food. In addition, the strong affinity of fat for lipophilic substances in the packaging can increase the release rate of these compounds compared to other food components (Etxabide et al. 2022).

### 2.1 Factors Related to Packaging Material

Different surfaces have different permeability properties against migrating compounds. The thickness, density, chemical structure, and permeability properties of the material are among the factors affecting the migration process. Although thick packaging reduces the migration rate, thin packaging can increase the amount of migration. In addition, the larger packaging surface area enhances the possibility of migration (Alamri et al. 2021; BRCGS 2017). The initial amount of migrants present in the packaging material is another critical factor that can influence the extent of migration (Alamri et al. 2021). Additionally, the presence of a barrier layer between the packaging and the food can play an important role in migration, as it can prevent or delay the transfer of substances.

A study investigating mercury migration into various food simulants evaluated four types of paper–plastic packaging: (A) printed, (B) unprinted, (C) printed with an aluminum foil layer, and (D) unprinted with an aluminum foil layer. The highest level of mercury migration was observed in the printed packaging (A), whereas the lowest was detected in the unprinted packaging that included an aluminum foil barrier (D). These findings suggest that printing components can enhance chemical migration, whereas aluminum foil serves as an effective barrier that significantly reduces it. The study emphasizes the importance of carefully considering the use of printing materials in packaging design and highlights the protective role of barrier layers such as aluminum foil in minimizing the transfer of harmful substances into food (Peng et al. 2020). Similarly, the structural characteristics of paper‐based packaging—such as porosity and density—have also been shown to significantly influence the migration of organic compounds. Triantafyllou et al. (2005) compared the migration behavior of 100% recycled paper (S1), characterized as a less dense and more porous structure, with 0% recycled paper (S2), which exhibited a denser and tighter structure. Their findings showed that the retention capacity of organic migrants was lower in S1 paper, leading to higher migration into the air phase. This suggests that S1 paper provides a more permeable environment for volatile organic compounds. In contrast, S2 paper retained organic substances more effectively within the paper matrix, limiting their transfer to the air phase. These results highlight the significant influence of physical properties such as porosity, density, and surface characteristics on the migration of organic compounds from paper to the air phase. Packaging materials that are less dense and more porous allow greater migration, whereas those with higher density and lower porosity act as barriers, limiting the permeability and thereby reducing chemical migration. Furthermore, the use of recycled materials has also been observed to contribute to increased migration levels. Additionally, repetitive use of packaging was also seen as a factor influencing migration. Bhunia et al. (2013) reported that repeated use led to increased migration of bisphenol A (BPA). In newborn baby bottles, BPA concentrations were 0.03 ppb at 40°C and 0.13 ppb at 95°C. However, after 10 uses, the concentration increased to 3.8 ppb, and this level persisted even after 100 uses. In parallel with concerns regarding reuse, the choice of packaging material itself—particularly the adoption of biodegradable alternatives—has also been studied in the context of migration safety. In this regard, Balaji and Immanuel (2022) investigated the migration behavior of biodegradable packaging films made from low‐density polyethylene (LDPE) blended with modified corn starch during a 6‐month storage period using potato chips and biscuits as test foods. Although factors such as food type, contact duration, and film thickness influenced migration levels, the detected migration in films with T1 (LDPE: modified starch 95:5) and T2 (90:10) compositions remained below the legal limits. These findings suggest that such biodegradable films can be safely used as food‐contact materials. In another study, chicken meats after cooked in oven bags belonged to five different brands (A, B, C, D, and E) were examined in terms of BPA migration. BPA was not detected in chicken meats cooked without oven bags, whereas varying levels of BPA were found in samples cooking with oven bags, depending on the brand (brands B, C, and D were undetectable, brand A 8.65 ng/g, and brand E 63.78 ng/g). These findings suggest that different plastic materials can significantly influence migration levels (Savaş et al. 2021).

### 2.2 Foodborne Factors

Characteristics such as the form of the food, its fat and moisture content, and its surface area are key factors that influence migration (BRCGS 2017). For example, the fat content in food can increase the movement of lipophilic substances from the package by allowing oil to transfer between the food and the packaging material. This process promotes the migration of these substances into the food. Additionally, oil's strong affinity for lipophilic substances in the packaging can increase their release rate compared to other food components (Etxabide et al. 2022). Xu et al. (2010) examined the effect of food's lipophilic properties on migration. They found that phthalate migration ranged from 1% to 14% in edible oil but remained below 0.35% in mineral water, highlighting the significant impact of oil content on migration. Baele et al. (2020) investigated chemical migration in paper and cardboard packaging and found that the fat content of the food had a significant impact on migration levels. The study revealed that higher levels of migration were observed in high‐fat foods such as chocolate (40% fat) and biscuits (8%–25% fat). Moderate levels were detected in foods with lower fat content, such as egg pasta (2.6%), wheat flour (1%), and rice flour (0.5%), whereas low levels of migration were found in wheat pasta (0.4%). These findings indicate that fat content is a critical factor influencing migration. Additionally, the volatility of contaminants was also noted to play a role in migration behavior. Therefore, when packaging high‐fat foods, it is essential to consider materials and solutions—such as barrier layers—that can effectively minimize chemical migration. Kubwabo et al. (2009) investigated the migration of BPA from plastic containers into water and ethanol. Their results showed that BPA migration into 50% ethanol at 40°C after 240 h (2.39 µg/L) was greater than migration into water (1.88 µg/L), demonstrating that migration rates vary depending on the medium. Additionally, foods with a high surface area‐to‐volume ratio, such as pasta, generally exhibit higher migration rates (BRCGS 2017). This phenomenon can be explained by the increased contact interface between the food and the packaging material, which facilitates more points of chemical exchange. As the available surface area in contact with the packaging increases relative to the volume of the food, so does the potential for migration. Consequently, the surface area‐to‐volume ratio is considered an important factor in migration assessments, especially for foods with irregular shapes or large surface exposure. From a safety standpoint, this highlights the importance of adapting packaging design and material selection according to the physical characteristics of the food product to minimize contaminant transfer.

### 2.3 Factors Related to Migrants

The characteristics of migrating substances, such as molecular size, structure, and volatility, significantly influence migration (Muzeza et al. 2023). Volatile compounds and smaller molecules tend to migrate at higher rates (Arvanitoyannis and Kotsanopoulos 2013; BRCGS 2017), whereas long‐chain molecules generally do not transfer into food (Nikova et al. 2022). Although migration is important in those with a molecular weight below 1000 Da, it is assumed that oligomers with a molecular weight below this size undergo gastrointestinal absorption in terms of risk assessment (Kato and Conte‐Junior 2021). This assumption highlights that, in addition to structural properties such as low molecular weight, factors like the absorption potential and biological effects of substances migrating into the body also play a critical role in risk assessment. Tegladza et al. (2023) examined the migration of methyl isocyanate (MIC), naphthalene (NAPH), and caprolactam (CAP) with varying molecular sizes and found that molecular size significantly affects migration. Their results demonstrated that smaller molecules migrate more readily, with MIC (57.05 g/mol) > NAPH (128.17 g/mol) ≈ CAP (131.16 g/mol). Similarly, Triantafyllou et al. (2005) compared compounds with varying molecular sizes, boiling points, and volatilities, including acetophenone, NAPH, benzophenone, dibutyl phthalate (DBP), and methyl stearate. They reported that compounds with higher molecular weights, higher boiling points, and lower volatility, such as DBP, methyl stearate, and benzophenone, adhered more to the packaging. In contrast, acetophenone and NAPH, with lower molecular weights, lower boiling points, and higher volatility, migrated more easily to the air, demonstrating their greater migration potential. In a study focusing on the impact of molecular weight on migration, the transfer of 13 different organophosphate esters (OPEs) from polypropylene (PP) packaging into high‐fat foods was investigated. The study was conducted at varying temperatures (25°C, 40°C, and 60°C), and the results showed that OPEs with lower molecular weights—particularly those below 300 Da—migrated more rapidly and in greater quantities. These findings underline the need for careful consideration when using such compounds in food contact materials. Additionally, the study highlighted the amplifying effects of temperature and fat content on migration levels, reinforcing the importance of selecting packaging materials and storage conditions that are appropriate for the specific type of food product (Miao et al. 2024).

### 2.4 Factors Related to Contact

Direct or indirect contact between food and packaging significantly impacts migration. Direct contact increases the rate of mass transfer, whereas indirect contact—where a gaseous layer exists between the food and the packaging—results in slower migration (Alamri et al. 2021). In a study comparing the effect of contact surface on migration, static and dynamic conditions for phthalate migration were evaluated. The findings showed that dynamic movement increased phthalate migration significantly due to the enhanced contact between the packaging and the food matrix (Xu et al. 2010). Another study by Page and Lacroix (1995) demonstrated the effect of contact area on migration. They placed smoked salmon, chicken breast, and minced meat—each with surface contact areas of 130, 72, and 27 µg/cm^2^, respectively—on polystyrene trays. Migration levels were reported as 220, 14, and 9.5 µg/g, respectively, showing that a larger contact area leads to higher migration levels.

Storage time is another critical factor influencing migration (Bhunia et al. 2013). Products with long shelf lives or nearing their expiration dates are more likely to experience increased migration. For instance, styrene levels in dairy products were observed to increase after cold storage exceeding 30 days (Kontou et al. 2021). Similarly, antimony and aluminum levels in water stored in polyethylene terephthalate (PET) bottles rose with extended storage times (Aghaee et al. 2014). In seafood packaged with PP and polyvinyl chloride (PVC) materials, phthalate ester levels also increased during prolonged storage (Alp and Yerlikaya 2019). On the basis of migration levels monitored over a 4‐month period, di‐(2‐ethylhexyl) phthalate (DEHP) was identified as the most prevalent migrating phthalate. The study demonstrated a clear relationship between migration and contact duration, showing that the amount of migrated chemicals increased in parallel with longer storage times. Therefore, it should be noted that foods with extended shelf lives may be more susceptible to higher levels of migration, emphasizing the importance of maintaining appropriate environmental conditions to preserve food quality and safety.

### 2.5 Environmental Factors

The temperature that the products exposed during cooking, storage, and processing affects migration. Although less migration occurs at freezing temperatures, the probability of migration increases as the temperature rises (Genualdi et al. 2014; BRCGS 2017). Estremera et al. (2023) examined how cooking time and temperature affect chemical migration using the sous vide technique. Migration increased significantly at 68°C after 250 min, highlighting the risks of prolonged high‐temperature exposure and the need to control thermal processing to reduce migration. Bhunia et al. (2013) studied BPA migration in four different canned foods and reported high levels of BPA transfer from the can lining to the food during processing at 121°C for 90 min. Similarly, a migration study conducted at 70°C and 100°C in two types of paper packaging (S1; less dense and more porous structure, S2; denser and tighter structure) revealed that migration levels increased significantly at the higher temperature for both packaging types and all tested substances (Triantafyllou et al. 2006). In a study comparing heating durations, oven heating and microwave heating were evaluated, and it was observed that microwave use caused less migration due to the shorter exposure to heat (İçöz and Eker 2016). Sapozhnikova et al. (2021) examined the impact of various cooking materials—including microwave trays, microwave oven bags, and conventional oven bags—on chemical migration, identifying a total of 74 different compounds. The migration levels followed the trend: microwave oven bags > microwave trays > oven bags. Microwave processing resulted in significantly higher migration compared to oven heating. Specifically, 20 compounds exhibited elevated migration levels in the microwave, whereas only 5 compounds were more prominent after oven use. The study emphasized that high‐temperature processes like microwaving can lead to the migration of a wide range of chemicals, highlighting the need for careful evaluation of food‐contact materials used in such applications. These findings reinforce the critical role of temperature in chemical migration and demonstrate that both increased heat and prolonged exposure duration significantly amplify migration rates. Consequently, the importance of maintaining appropriate environmental and processing conditions for packaged foods is further underscored.

Cooking techniques also play a role in migration. Fierens et al. (2012) reported that levels of diisobutyl phthalate (DiBP) decreased during boiling and frying but remained unchanged during steaming. Tsumura et al. (2001) compared DEHP migration in raw chicken at different stages, finding that migration levels were highest after packaging (16.9 mg/kg), followed by frying (13.1 mg/kg), and were minimal in raw chicken (0.08 mg/kg). Furthermore, several studies on DEHP have reported significant reductions in migration levels following drying processes (Wang et al. 2023). These findings demonstrate that cooking methods can have varying effects on chemical migration and that consumers may be able to minimize associated risks by selecting appropriate preparation techniques.

Another case influenced by factors such as storage conditions and temperature is the formation of hydroxymethylfurfural (HMF). Although HMF is not classified as a chemical that migrates directly from packaging, it is a heat‐induced compound formed during food processing and poses significant toxicological risks. These risks include hepatotoxicity, nephrotoxicity, neurotoxicity, reproductive and developmental toxicity, genotoxic and mutagenic effects, cytotoxicity, and enzyme inhibition (Farag et al. 2020). HMF is found in high concentrations, particularly in plant‐based processed foods, including coffee, cocoa, baby food, pasta, dried fruits, baked goods, milk, honey, and vinegar (Michalak et al. 2016; Capuano and Fogliano 2010; Farag et al. 2020). In a study conducted by Michalak et al. (2016), 90 food samples—including baby formulas, infant biscuits, cocoa, coffee, and bread products—were analyzed to evaluate the effects of storage duration and temperature on HMF concentration. The results showed a general increase in HMF levels with longer storage times and higher temperatures. Notably, a significant rise was observed in coffee substitutes: a product with an initial HMF concentration of 397.0 mg/kg reached 432.0 mg/kg after 6 months and 469.0 mg/kg after 12 months at 4°C. When stored at 25°C, HMF levels rose to 456.0 and 524.0 mg/kg at the same intervals. In addition to the effects of storage time and temperature, the study also found that high sugar content and an aw (water activity) level of approximately 0.4 significantly contributed to HMF formation. The results underline the impact of environmental conditions and food composition on HMF concentration and reinforce the importance of appropriate storage practices in ensuring food safety.

## 3 Packaging Materials

Today, a wide variety of materials and alloys are used in packaging, including plastic, cardboard, metal, and glass, each with distinct properties. The choice of packaging material is influenced by various factors such as the type, composition, and physical characteristics of the food; desired storage and shelf life; costs; and potential interactions between the packaging and the food. In addition to these considerations, environmental concerns and sustainability have also become critical factors shaping preferences in the packaging industry.

### 3.1 Plastic

Plastic can be processed to meet specific needs and produced in various forms, such as flexible, lightweight, soft, rigid, permeable, or as a barrier material. These versatile properties make plastic an indispensable material in the packaging industry, positioning it as the leading choice for an ideal, economical, and efficient material (Keskin and Koçoğlu 2022). Although its usability makes it a top choice, concerns regarding the health risks posed by the migration of its components into food have also placed it under scrutiny. Plastics are composed of various polymers and additives, and their types, monomers, and applications are summarized in Table 1 (Muzeza et al. 2023; Keskin and Koçoğlu 2022; Karakoç and Dikmen 2024).

**TABLE 1:** Commonly used plastics in packaging production.

Material type	Usage area	Monomer
Polyethylene terephthalate (PET)	Water, soft drink, alcohol, and edible oil bottles; fruit/vegetable packages	Ethylene terephthalate
High density polyethylene (HDPE)	Milk and juice boxes; oil bottles; plastic bags	Ethylene
Polyvinyl chloride (PVC)	Meat trays; bottles containing liquid foods (oils, vinegars, and beverage foods); flexible films for wrapping solid foods (fresh fruits, cheese, meat, and vegetables); coatings in metal cans, and lunch boxes	Vinyl chloride
Low density polyethylene (LDPE)	Shrink packaging; stretch films	Ethylene
Polypropylene (PP)	Margarine tubs; microwaveable meal trays; lunch boxes; sweets and snack wrappers; dairy products; refrigerated containers; bottle caps packages of ketchup, syrup, and yoghurt	Propylene
Polystyrene (PS)	Disposable coffee cups; plastic food boxes; containers for yoghurt, ice cream, fruit juice, and cheese; egg cartons; biscuit trays; meat packages, dairy products; low‐fat foods such as coffee, ice cream, yogurt, cream; honey, eggs, and fruit packages	Styrene
Polycarbonate (PC)	Recyclable beverage containers; ovenable frozen‐food trays; ready‐to‐eat meal boxes	Bisphenol A
Polyamide (PA)	Vacuum‐packaged cheese and freshly processed meats, frozen foods	Caprolactam

Plastics can also release substances known as microplastics, which result from the breakdown of plastic materials due to environmental factors, and with further degradation, they can transform into even smaller nanoplastics. Thus, plastics that have degraded into extremely small particles can easily disperse into various environmental media such as air and water. This increases the likelihood of human exposure and significantly elevates the risk of ingestion through the food chain. These particles are ingested in significant quantities through everyday items such as packaged foods, takeout containers, and plastic water bottles (Jadhav et al. 2021; Du et al. 2020). The three most significant factors influencing microplastic migration are temperature, mechanical stress, and the age of the packaging material. These factors increase microplastic ingestion through food, intensifying the potential health risks associated with plastics (Jadhav et al. 2021). Du et al. (2020) reported that takeout containers contain significant amounts of microplastics. Individuals who consume meals from these containers 4 to 7 times per week may ingest between 12 and 203 microplastic particles. In another study focusing on drinking water, microplastic contamination was detected in 93% of bottled water samples from 11 different brands. Furthermore, the microplastic concentration in bottled water was found to be double that of tap water (Mason et al. 2018). The presented information demonstrates that the consumption of take‐away meals and bottled water packaged in plastic significantly contributes to microplastic exposure, underscoring once again the importance of choosing safe and appropriate packaging materials. In another study analyzing mineral water, researchers examined glass, reusable PET, and single‐use PET bottles from various brands for the presence of microplastics, taking into account both new and used bottles. The results revealed that although microplastics were detected in almost all of the bottles, the lowest amount was detected in the single‐use PET bottles (2649 microplastics/L), followed by the reusable PET bottle (4889 microplastics/L). In addition, although similar values were detected in newly produced single‐use and reusable PET bottles, these amounts were significantly higher in old bottles (Oßmann et al. 2018). Hernandez et al. (2019) examined the impact of temperature on microplastic release, and they demonstrated that brewing a single plastic tea bag at 95°C released approximately 11.6 billion microplastic particles and 3.1 billion nanoplastic particles into the beverage. However, human exposure to microplastics is not limited to food; atmospheric microplastic pollution also contributes to this exposure (Du et al. 2020). Today, plastic is an extensively used material across all areas of life and has become an inseparable part of the food industry. Although it may seem impossible to completely separate plastics from the food sector in practice, making careful choices can help minimize exposure to the harmful chemicals contained in plastics. The European Parliament has adopted regulations to reduce packaging waste by setting binding reduction targets (5% by 2030, 10% by 2035, and 15% by 2040) and mandating minimized packaging volume and weight, with a particular focus on reducing plastic waste. Effective January 1, 2030, the legislation bans specific single‐use plastic packaging types, including those for fresh produce, condiments, and toiletries, as well as very lightweight plastic bags. Additionally, the regulation prohibits the use of “forever chemicals” (Perfluoroalkyl substances [PFASs]) above set thresholds in food‐contact packaging to protect consumer health (European Union 2025).

### 3.2 Biodegradable Packaging

Biodegradable polymers have emerged in order to reduce the risk of plastic. These are biodegradable by the action of microorganisms such as bacteria, fungi, and algae. These bioplastics, which have similar properties to plastics such as PET, PP, and PE, include PLA (polylactic acid), PHA (polyhydroxyalkanoates), PBS (polybutylene succinate), PBAT (polybutylene adipate terephthalate), starch blends, and others (Kirwan and Strawbridge 2003; Shaikh et al. 2021).

Although biodegradable polymers make up only 1% of all plastics, they are considered environmentally friendly because they are derived from renewable raw materials and agricultural waste. However, when evaluated in terms of migration into food and potential toxicity in humans, they may pose risks due to the presence of various substances, including additives, antioxidants, and stabilizers used in their production (Shaikh et al. 2021). Some researchers consider bioplastics as safer alternatives to conventional plastic polymers (Jadhav et al. 2021). However, others regard them as a significant concern due to the limited knowledge about their chemical safety and the potential for increased exposure to their released chemicals as their usage grows (Zimmermann et al. 2020). In their study, the researchers demonstrated that bioplastics and plant‐based materials are not necessarily safer than conventional plastics, as commonly assumed. These materials exhibited a broad chemical diversity, and many were found to be toxic in vitro assays. The study highlights the need for comprehensive chemical analyses and risk assessments before promoting such materials as safer alternatives for food packaging.

### 3.3 Metal

Various metals, including copper, silver, brass, bronze, lead, tin, and zinc, find applications in the packaging industry. However, steel and aluminum are the most widely used metals due to their numerous advantages. The growing demand for metal in food packaging can be attributed to properties such as safety, hygiene, mechanical durability, impermeability, and excellent barrier protection against gases, moisture, and light (Piergiovanni and Limbo 2016). Additionally, metals exhibit resistance to heat, insects, and rodents, while also allowing for surface polishing and custom design (Page et al. 2011).

Despite these benefits, certain disadvantages of metals in food packaging cannot be overlooked. These include their relatively heavy weight, difficulties in opening, and the high cost of metal production. Moreover, metals require a protective layer called “lacquer” to prevent interactions with food (Deshwal and Panjagari 2019). Tin, commonly used in metal packaging, is prone to corrosion upon contact with food. If the coating is damaged, tin may leach into the food, potentially posing serious health risks when its concentration exceeds the threshold of 730 ppm (Boogaard et al. 2003). Similarly, aluminum, under corrosive conditions, can migrate from packaging into food, accumulating in the human body and potentially causing adverse health effects with prolonged exposure (Arvanitoyannis and Kotsanopoulos 2013). To mitigate oxidation and improve adhesion properties, metal packaging is often chrome‐plated. However, in cases of corrosion, chromium can also leach into food, presenting significant food safety risks due to its toxic effects (Kim et al. 2008). In addition, plastic polymer coatings used in aluminum and steel cans also pose problems arising from plastic polymer migration (Cooper et al. 2011).

### 3.4 Paper/Cardboard

The environmentally friendly, easily recyclable, and sustainable nature of paper and cardboard makes them among the most preferred materials for packaging. Cardboard is formed by combining papers of different thicknesses and offers more advanced properties compared to paper. Today, paper and cardboard are frequently used for various applications, including pizza boxes and other fast‐food packaging, beverage containers, baking papers, and microwave popcorn bags (Deshwal et al. 2019).

Substances that can migrate from paper and cardboard packaging include mineral oils, dyes, phthalates, adipates, polyfluorinated compounds, and adhesives (Fierens et al. 2012). Due to their weak barrier properties, they often require coating with plastic polymers, which can lead to similar migration issues as those seen with plastics. Moreover, recycling paper and cardboard does not eliminate these migratory substances. Instead, it can result in mineral oil concentrations exceeding acceptable limits, with mineral oil migration being identified as a primary culprit in food contamination (Biedermann and Grob 2010). Additionally, materials containing recycled cellulose have been reported to contribute to contamination with phenol‐class compounds, such as polycyclic aromatic hydrocarbons (PAHs) and BPA (Vinggaard et al. 2000). Although paper and cardboard are often viewed as safer alternatives to plastic, recycled forms of these materials may still pose notable health risks due to their higher potential for chemical contamination. Moreover, their inadequate barrier properties represent an additional concern that should be carefully considered when selecting these materials for food packaging applications.

### 3.5 Glass

Glass is one of the oldest known materials. Beyond its use in preserving and packaging food, it holds value for the future as it helps protect the environment and conserve natural resources. Glass is not composed of a single substance but rather a mixture, which is tailored on the basis of its intended use, desired color, and specific properties (González‐López et al. 2023). Glass packaging has both positive and negative attributes. Among its advantages are its durable, robust, and inert structure. It is suitable for sterilization and cleaning processes and can be manufactured in various sizes, shapes, and colors to meet diverse needs. Its transparency allows the contents to be easily seen. Additionally, its reusability and recyclability make it environmentally significant. Glass does not wear out or degrade over time, making it ideal for vacuum sealing, filling, and capping processes. It is impermeable to gases, water vapor, odors, and liquids (Kim et al. 2013). However, glass also has drawbacks. Its transparency allows light to pass through, which can impact the contents. Its fragile structure, higher air pollution impact (three times more than plastic packaging), high transportation and production costs, and heavier weight are considered disadvantages (Yaris and Sezgin 2017). Research shows that silica and alkali are the primary components that can leach from glass. However, these components do not significantly affect the organoleptic properties of food (Arvanitoyannis and Kotsanopoulos 2013). Contamination from lead and cadmium migration is minimal, as these substances are rarely used in glass containers (De Fátima Poças and Hogg 2007). Despite advancements in production technology and increased recycling rates, migration from glass packaging to food occurs at very low levels (Arvanitoyannis and Kotsanopoulos 2013). Additionally, unlike other packaging materials that require an extra protective layer to ensure food safety, glass packaging provides inherent protection. This further strengthens its reputation as a safe option in terms of migration (Schrenk 2014).

### 3.6 Recycled Packaging

Recycled packaging materials are one of the methods of ensuring sustainability in packaging. These materials include molded pulp, corrugated cardboard, cardboard, newsprint, glass, plastics, and some metals. Among them, paper and cardboard are always the most recycled packaging materials (EPA 2020). However, in the process of preparing recycled paper or cardboard, many chemicals that can adversely affect human health are used for various stages such as pulping, bleaching, and sizing (Deshwal et al. 2019). The use of this recycled material as packaging poses a significant problem due to the risk of migration through the gas phase, even if there is no direct contact with food (Rothenbacher et al. 2007). In addition, adhesives and printing inks are major sources of migration‐related contamination, especially in recycled packaging materials, where contamination levels tend to be higher. The migrating substances primarily consist of organic compounds such as hydrocarbons, alcohols, glycol ethers, ketones, and esters. These substances can transfer to food either through direct contact or by permeating through the empty spaces within the packaging, even without direct contact (Gupta et al. 2024). Studies have reported that contaminants in recycled packaging materials have increased in number and range recently (Etxabide et al. 2022). Moreover, in 2024 the European Parliament adopted the Packaging and Packaging Waste Regulation (PPWR), replacing Directive 94/62/EC, which mandates all packaging to be recyclable by 2030 and bans certain single‐use plastics from 2030 (European Union 2024a). These measures emphasize the need for a comprehensive redesign of packaging materials to address the increased risk of chemical migration, particularly in recycled materials used for food packaging. Prioritizing safer packaging designs and incorporating innovative barrier technologies is essential to minimize contamination risks and ensure food safety, given the growing variety of contaminants found in recycled materials.

## 4 Migrants Posing Risks to Public Health and Their Presence in Food

Substances that can migrate from food packaging are classified as intentionally added substances (IAS) and non‐intentionally added substances (NIAS), including the migrants they generate. IAS are typically derived directly from the packaging itself, originating from substances intentionally used during the manufacturing process, such as monomers, plasticizers, antioxidants, and photoinitiators. In contrast, NIAS are compounds that are not intentionally added during the production process and are not planned to come into contact with food (Schrenk 2014). When these compounds come into contact with food, migration may occur as a result of the interaction between the packaging and the foodstuff. The ingestion of these migrants through food can lead to various health issues depending on the toxicity of the substance, its concentration, and an individual's sensitivity (Etxabide et al. 2022). Studies on NIAS such as nonylphenol (NP)—a compound that can form as a result of degradation and oxidation processes—have shown that it may lead to thyroid dysfunction. Furthermore, perinatal exposure to NP has been associated with an increased risk of obesity in offspring (Ong et al. 2020). Ibarra et al. (2018) analyzed 34 plastic‐packaged foods (potato snacks, corn snacks, cookies, and cakes) and identified over 40 substances, including plasticizers, UV absorbers, antioxidants, and fatty acid derivatives. Acetyl tributyl citrate (ATBC) was the most prevalent, found in 94% of samples and showed higher migration in fatty foods. Although phthalates were detected, they generally remained below migration limits. These results also indicate that fatty foods are particularly prone to chemical migration, further emphasizing the importance of selecting packaging materials on the basis of the type of food they will contain. In another study, phthalate migration (DIBP and DBP) during cooking chicken in plastic bags with and without spices was investigated. The amount of DIBP and DBP detected was very low (0.06–0.24 µg/kg) when chicken was cooked in plastic bags without spices, but these levels increased significantly when spices were added (0.01–4.57 µg/kg). Although levels remained below regulatory limits, spices were identified as a significant source of migration (Moreira et al. 2015). As a result of the migration of these substances, one of the most significant problems encountered is disruption of the endocrine system. Additionally, they have been linked to various other health issues and have also been reported to cause lung tumors in rodents (Schrenk 2014).

Plastic materials top the list of the most common interactions between food and packaging. Low molecular weight substances such as plasticizers, antioxidants, and monomers can migrate even from ordinary plastic bags, causing contamination. Among these, styrene and BPA are notable migrants associated with significant health issues, such as cancer and reproductive problems (Bansal and Gupta 2020; EPA 1994). On the basis of cancer research and genotoxicity findings, styrene has been identified as a carcinogenic compound, particularly associated with an increased risk of cancers related to the lymphatic and hematopoietic systems (Huff and Infante 2011). BPA, classified as an endocrine disruptor, negatively impacts endocrine system functions, adversely affecting development and various vital processes in the body (Hunt et al. 2003). Wang et al. (2019) reported that BPA exposure is associated with glucose homeostasis disorders—a condition that often precedes diabetes—in middle‐aged and older women. Endocrine‐disrupting chemicals (EDCs) are among the most concerning migrants due to their potential for transgenerational effects and irreversible health risks. Although exposure to these chemicals poses risks for adults, they are particularly harmful to infants, children, and pregnant women (Hass et al. 2019; Butterworth 2009; Ong et al. 2020). In a study investigating the effects of prenatal EDC exposure, Tanner et al. (2019) found that these substances negatively impact neurodevelopment, with male children exhibiting reduced cognitive function by the age of 7. Exposure during the prenatal and early postnatal periods may lead to health outcomes that manifest in childhood or adulthood. These long‐term and potentially heritable effects include behavioral changes, altered timing of puberty, reduced fertility and sperm quality, and an increased risk of breast, prostate, and testicular cancers (Hass et al. 2019).

In addition to BPA, phthalates, which also belong to the group of plasticizers, are classified as EDCs (Schrenk 2014). Among them, DEHP is the most widely used high‐molecular‐weight phthalate in food‐grade plastic packaging, and consequently, it is the most commonly detected phthalate in foods (Wenzl 2009). As these compounds are not chemically bound to the polymer matrix, they are highly prone to migration from packaging materials into food. This is especially true for lipophilic foods such as meat, dairy products, and vegetable oils, into which phthalates migrate at higher levels due to their fat‐soluble nature (Lovrenović et al. 2020). Once ingested through the diet, phthalates not only exert endocrine‐disrupting effects but are also associated with a range of other adverse health outcomes. These include toxicity to the reproductive system and tumor formation (Lovrenović et al. 2020; Schrenk 2014). Studies have reported that BBP (benzyl butyl phthalate), DBP, DiBP, DIDP (diisodecyl phthalate), DiNP (diisononyl phthalate), and DEHP can cause reproductive toxicity in male mammals. Furthermore, DBP and DiBP have been linked to preterm birth, low birth weight, and reduced fertility (Wormuth et al. 2006; Koch et al. 2012). It should also be noted that although dietary intake is a major route of exposure, the fact that these compounds are not chemically bonded makes their release into the environment easier, enabling additional exposure pathways such as inhalation (Lovrenović et al. 2020).

Even trace amounts of these chemicals are reported to pose threats to human health (Bansal and Gupta 2020). Furthermore, among the 906 chemicals associated with plastic food packaging, 63 have been identified as hazardous to human health, 68 as environmentally dangerous, 7 as persistent, bioaccumulative, and toxic (PBT), 15 as endocrine disruptors, and 34 as potential endocrine disruptors (Groh et al. 2018). However, current knowledge about the impacts of both known and unknown chemicals, including monomers used in plastic food packaging, on human and environmental health remains insufficient. Some known migrants that may migrate from packaging and the health risks they pose are listed in Table 2.

**TABLE 2:** Some toxic migrants and their effects on health.

Immigrant	Health risk	Type of packaging that can be found	Source
Bisphenol A (BPA)	– Breast, ovarian, uterine, prostate and testicular cancer – Estrogenic effect – Disrupting the hormonal balance by affecting various receptors – Disrupting the thyroid axis – Hyperactivity, neurodevelopmental disorders, and metabolic disorders resulting in type 2 diabetes – ‐Infertility – Influencing intestinal permeability – Breast and prostate cancer – Disrupting oxidative balance – Increase in hydrogen peroxide and lipid peroxidation – Effect on the organogenesis of the kidneys, brain and testicles in the fetus – Anxiety in childhood – Cardiovascular function disorders – Mitigating the effect of DNA methylation	Plastic Paper/Recycled paper, cardboard Metal	Jones and Watson (2012), Peretz et al. (2014), Vinggaard et al. (2000), Herrero et al. (2020)
Phthalates	– Antiandrogenic effect – Dysfunction in the thyroid axis – Reproductive disorders: low sperm count, shortening of anogenital distance in men, increased risk of premature birth – Increase in the level of estrogen in pregnant women – Birth defects – Asthma – Hypospadias – Cryptorchidism – Neurobehavioral problems – Cardiovascular diseases – Immunological diseases	Plastic Paper Metal	Wójtowicz et al. (2018), Zelko et al. (2021), Zhu et al. (2020), Rudel et al. (2011), Muncke et al. (2023), Muzeza et al. (2023), Fierens et al. (2012), Herrero et al. (2020)
Styrene	– Reproductive toxicity – Developmental toxicity – Hepatotoxic effect – Chromosomal abnormalities – Carcinogenic effect – Mucous membrane irritation – Effect on the respiratory system – Ocular and nasal irritation – Gastrointestinal effect – CNS dysfunction, neurotoxicity – Effect on certain renal enzyme functions and hematological parameters		
	– Lung tumors – Stimulation of cell replication, promotion of cell proliferation and formation of cytogenetic damage		
Caprolactam	– Effect on CNS, neurasthenia syndrome – Neurological, gastrointestinal and cardiovascular effects and dermatological and immunological changes in long‐exposed workers	Plastic	Zhu et al. (2020), Ianneo (2009)
Vinyl chloride	– Hepatotoxic, nephrotoxic, pulmoner toxic – Effects on the spleen, nervous system, and blood – ‐Cancer – Fatty liver – Increased risk of alcoholic hepatitis	Plastic	Zelko et al. (2021)
Dioxin	– Increase in metabolism – Suppression of thyroxine concentration – Decrease in blood insulin and glucose levels – Increase in serum gastrin – Infertility, fetal loss – Decrease in spermatogenesis – Decrease in circulating androgens – Endometriosis – Inhibition of growth factor and vitamin A expression – Ovarian dysfunction	Plastic Paper	Dufour et al. (2018), Ackermann et al. (2006)
Paraben	– Estrogenic and antiandrogenic activities – Impact on fertility – Disrupting postnatal growth in boys – Weight gain – Abnormal sperm production – Decrease in testosterone level – Cancer – Weakness in enzyme activities that affect hormone metabolism	Plastic Metal Cardboard	Jurewicz et al. (2020), Kim and Chevrier (2019), Herrero et al. (2020)
Perfluoroalkyl substances (PFASs)	– Decrease in thyroid hormones – Effect on thyroid metabolism in pregnant women and children – Increase in hyperactivity – Developmental and immune toxicity – Cancer (kidney and testicular cancer) – Weight gain – Liver degeneration – Influence on the development of the nervous system – Suppression of the immune response – Decrease in birth weight	Plastic Paper	Genthe and Steyn (2008), Stroheker et al. (2004), Boas et al. (2010), Wolff et al. (2014), Karakoç and Dikmen (2024), Fierens et al. (2012)
	– Diabetes – Decrease in birth weight – Diabetes		
Heavy metals	Cadmium: estrogenic effect, breast cancer Aluminum: negative impact on brain and bone health with high aluminum exposure, estrogenic effect, breast cancer Mercury: thyroid gland functions and impairment on the hypothalamic‐pituitary‐adrenal axis, breast cancer Lead: cellular enzyme inhibition, effect on erythrocyte membrane stability, functional disorders in peripheral nerves and skeletal development, estrogenic effect, breast cancer Chromium: carcinogenic and mutagenic effect	Metal Recycled paper	Shimizu et al. (2018), Arvanitoyannis and Kotsanopoulos (2013), Skrzydlewska et al. (2003)
Benzophenone	– Endocrine disruptor in infants and pregnant women – Estrogenic effect – Genotoxide – Carcinogenic effect	Paper/Recycled paper	Muncke (2009, 2010)
Nitrozamine	– Genotoxide – Carcinogenic effect	Paper	Tricker and Preussmann (1991)
Naftilamin, benzidin, 4‐aminobifenil	– Mixed cans	Recycled paper	Muncke (2010)

The migration of chemical substances from food packaging into food not only affects food safety and quality but also poses a significant risk to public health due to potential toxicity (Gupta et al. 2024). Migration tests, performed with real food samples or food simulants, are essential for determining the types and concentrations of chemical compounds that consumers may be exposed to. In addition, migration levels depend not only on the composition of the packaging material but also on various factors such as the food matrix, temperature, duration of contact, and environmental conditions (Arvanitoyannis and Kotsanopoulos 2013).

Chemicals used in food packaging are regulated to ensure they remain within safe migration limits, thus minimizing potential health risks. These specific migration limits (SMLs) are periodically updated on the basis of scientific research and risk assessments, which are subsequently incorporated into regulatory frameworks. BPA, in particular, has been under scientific examination for years due to its endocrine‐disrupting effects, leading to increasingly strict usage restrictions. In 2023, the European Food Safety Authority (EFSA) lowered the tolerable daily intake of BPA to 0.2 ng/kg body weight and declared that current exposure levels pose a health risk for all age groups (EFSA CEF Panel 2023). Consequently, in 2024, the European Union Member States approved a ban on the use of BPA, and the commission officially banned the use of BPA in food contact materials in January 2025 (European Union 2024b).

The EU regulation on plastic materials and articles intended to come into contact with food establishes migration limits for various substances to protect food safety. Among these, the permitted migration limits include 0.6 mg/kg for benzophenone, 15 mg/kg for CAP, and 1 mg/kg for aluminum. For phthalates, the specific limits are set at 0.6 mg/kg for DEHP, 0.12 mg/kg for DBP, and 6 mg/kg for BBP (European Commission 2011). However, analyses of commercially available food products have shown that certain chemicals from packaging materials can be detected in food at varying concentrations. For instance, chemicals used in plastic containers, cans, bottles, and packaging films have been found to migrate into food. This phenomenon is particularly prominent in fatty, acidic, or hot foods, increasing the likelihood of unintentional consumer exposure (Yenidoğan et al. 2023). Various studies have identified the presence of substances such as BPA, styrene, phthalates, benzophenone, and parabens in commercially available food products, highlighting potential health risks if migration limits are exceeded. Migrants detected in commercially available foods and their amounts are given in Table 3.

**TABLE 3:** Migrants detected in commercially available foods and their concentrations.

Migrant	Package material	Food	Concentration	Country	Reference
BPA	Can	Canned tuna in oil	<LOQ‐409 µg/kg	Spain	Gálvez‐Ontiveros et al. (2021)
		Canned sweet corn	42.7 µg/kg		
	Plastic	Chocolate doughnuts	82 µg/kg		
		Burger bun	1.2 µg/kg		
		Pizza (cooked ham and cheese)	4.3 µg/kg		
		Flavored yoghurt	60.85 µg/kg		
	Plastic + foil + paperboard	Chips (sour cream and onion)	8.8 mg/g		
	Undefined	Canned creamed soups	22.2 µg/kg	Canada	Cao et al. (2011)
		Canned peas	16.8 µg/kg		
		Rye bread	1.73 µg/kg		
		Canned corn	83.7 µg/kg		
		Prepared sandwiches	1.24 µg/kg		
		Cheese	2.24 µg/kg		
	Undefined	Canned cut green beans	5.60 µg/kg	United States	Lorber et al. (2015)
		100% vegetable juice cocktail	0.41 µg/kg		
		Frozen mixed vegetables	0.31 µg/kg		
		Fresh peach	0.24 µg/kg		
		Canned 98% fat free chicken breast	5.70 µg/kg		
		Canned corned beef	1.64–1.73 µg/kg		
	Plastic	Coffee drink	0.3–1 µg/kg	Japan	Sajiki et al. (2006)
		Cookies	1–14 µg/kg		
		Vegetable soup	3 µg/kg		
		Fresh beef	0.2 µg/kg		
	Can	Tuna	2–23 µg/kg		
		Chicken	4 µg/kg		
		Beef	9–10 µg/kg		
		Tomato soup	48 µg/kg		
		Mushrooms	4 µg/kg		
	Paper	Yoghurt	0.3 µg/kg		
		Cream	0.2–1 µg/kg		
	Can	Bean	85.08–1858.71 µg/kg	Turkey	Sungur et al. (2013)
		Tomato paste	21.86–109.71 µg/kg		
		Tuna	102.18–550.54 µg/kg		
		Garnish	66.18–366.80 µg/kg		
		Peas	28.88–303.72 µg/kg		
		Corn	78.16–230.84 µg/kg		
	Paper	Cream	43.84–112.26 µg/kg		
		Juice	40.27–82.55 µg/kg		
		Milk	81.09–156.22 µg/kg		
		Pudding	36.48–554.69 µg/kg		
	Glass	Mushrooms	19.22–339.21 µg/kg		
		Pepper paste	nd‐177.66 µg/kg		
		Pickle	33.54–290.76 µg/kg		
	Can	Orange juice	3.96 µg/L	Belgium	Geens et al. (2010)
		Iced tea	0.88 µg/L		
		Black olives	21.4 µg/kg		
		Anchovy	0.9 µg/kg		
		Sausages	26.7 µg/kg		
	Glass	Anchovy	0.67 µg/kg		
		Sausages	0.86 µg/kg		
		Pineapple	1.28 µg/kg		
		Corn	0.94 µg/kg		
	Plastic	Green olives	0.24 µg/kg		
		Pineapple	0.11 µg/kg		
		Vegetable soup	0.12 µg/kg		
Phthalate	Paperboard	Semolina powder	DiBP: 444–1796 µg/kg DnBP: 86.6–99 µg/kg DEHP: nd‐<LOQ	Germany	Gärtner et al. (2009)
		Oat flakes	DiBP: 34.6 µg/kg DnBP: <LOQ DEHP: nd		
		Baby rice cereal (2.4% fat)	DiBP: 67.7 µg/kg DnBP: <LOQ DEHP: nd‐<LOQ		
		Baby rice cereal (1% fat)	DiBP: 944 µg/kg DnBP: 100 µg/kg DEHP: nd		
	PET, glass or tinplate	Olive oil	DEHP: 1262 µg/kg DINP: 2884 µg/kg DBP: 360 µg/kg	Italy	Nanni et al. (2010)
		Sunflower oil	DEHP: 134 µg/kg DINP: 971 µg/kg DBP: 35 µg/kg		
		Corn oil	DEHP: 81 µg/kg DINP: 2982 µg/kg DBP: 23 µg/kg		
	Undefined	Green tea	DMP: 0.073–0.286 mg/kg DEP: 0.362–0.608 mg/kg DiBP: 0.109–0.742 mg/kg DBP: 0.443–0.872 mg/kg DEHP: 0.047–0.085 mg/kg	China	Du et al. (2015)
		Black tea	DMP: 0.028–0.515 mg/kg DEP: 0.336–0.936 mg/kg DiBP: 0.204–0.840 mg/kg DBP: 0.093–1.730 mg/kg DEHP: 0.031–0.164 mg/kg		
	Plastic	Lettuce	DEP: 24.3 µg/kg DBP: 13.8 µg/kg DEHP: nd	Spain	Cacho et al. (2012)
		Arugula	DEP: 51 µg/kg DBP: nd DEHP: nd		
		Parsley	DEP:8.0 µg/kg DBP: nd DEHP: 22 µg/kg		
		Chard	DEP: nd DBP: nd DEHP: 27 µg/kg		
	Plastic	Green tea	DMP: 0.22 µg/kg DEP: 2.96 µg/kg DiBP: 107 µg/kg DBP: 79.3 µg/kg DEHP: 452 µg/kg	China	Guo et al. (2012)
		Sausage	DMP: nd‐1.25 µg/kg DEP: 0.74–2.43 µg/kg DiBP: 4.68–12.8 µg/kg DBP: 6.99–14.6 µg/kg DEHP: 82.3–98.3 µg/kg		
		Coffee powder	DMP: 0.29 µg/kg DEP: nd DiBP: 8.49 µg/kg DBP: 14.4 µg/kg DEHP: nd		
		Candy	DMP: 0.23–20.5 µg/kg DEP: 0.31–3.14 µg/kg DiBP: nd‐76.2 µg/kg DBP: nd‐83.3 µg/kg DEHP: nd‐172 µg/kg		
		Fresh meat	DMP: 0.36–2.54 µg/kg DEP: 0.67–2.33 µg/kg DiBP: 0.37–42.6 µg/kg DBP: 0.73–13.2 µg/kg DEHP: 36.8–184 µg/kg		
		Rice	DMP: nd‐0.41 µg/kg DEP: 0.02–1.45 µg/kg DiBP: 1.82–70.8 µg/kg DBP: 1.48–99.0 µg/kg DEHP: 14.0–378 µg/kg		
Styrene	Polystyrene	Biscuits	37.9 µg/kg	Greece	Kontou et al. (2021)
		Soft cheese	1.6 µg/kg		
		Chicken burgers pre‐baked (homogenized)	67.5–160.1 µg/kg		
		Yoghurt from cow milk	5.6 µg/kg		
		Lettuce ready‐to‐eat salad	1.3 µg/kg		
	Polystyrene	Rice pudding	4.8 µg/kg	Germany	Guazzotti et al. (2023)
		Low‐fat curd cheese	4.5 µg/kg		
		Cream fine for cooking	13.8 µg/kg		
		Fruit (strawberry) yoghurt (with 9% fruit content)	8.0 µg/kg		
		Buttermilk	5.5–9.8 µg/kg		
	Polystyrene	Yoghurt	3.71 µg/kg	United States	Genualdi et al. (2014)
		Raw chicken	2.58 µg/kg		
		Bakery croissants	44.2		
		Noodle soup	4.33 µg/kg		
		Chewing gum	10.5–21.9 µg/kg		
		Chocolate candies	38.8 µg/kg		
Benzophenone	Cardboard	Gravy granules for meat	0.01 mg/kg	United Kingdom	Anderson and Castle (2003)
		Candy sticks	0.01 mg/kg		
		Basmati and wild rice	1.1 mg/kg		
	Cardboard	Instant noodles	15–89 µg/kg	Germany	Jung et al. (2013)
	Printed plastic foil	Wheat starch	429–531 µg/kg		
	Plastic foil	Rice paper	7530 µg/kg		
		Crunchy chocolate muesli	3413 µg/kg		
	Plastic foil; viewing window	Oriental sweet (kadaifi) (frozen)	1747 µg/kg		
	Plastic tray and foil	Cocoa coated cookie	1500 µg/kg		
	Plastic viewing window	Couscous	867–1559 µg/kg		
	Polycoupled cartons	Chocolate milk	9.98–39 µg/L	Italy	Sagratini et al. (2008)
		Skim milk	12.6–14 µg/L		
		Apple juice	9.9 µg/L		
		Red wine	5.5–217 µg/L		
	Carton	Milk	2.84–18.35 µg/kg	China	Shen et al. (2009)
Caprolactam	Multilayer polyamide	Poultry breast	21.8–25.4 mg/L	Brazil	Bomfim et al. (2010)
		Bologna sausage	7.9–23.2 mg/L		
		Ham	10.4 mg/L		
	Nylon	Liver pate	11 mg/kg	United Kingdom	Bradley et al. (2004)
		Polony sausage	13 mg/kg		
		Black pudding	2.8 mg/kg		
		White pudding	3.1 mg/kg		
Heavy metals	Metal cans or glass jars	Canned tuna	Pb: 0.159 mg/kg Cd: 0.012 mg/kg Hg: 0.048 mg/kg	Italy	Russo et al. (2013)
	Undefined	Milk	Pb: 1.99 µg/L Cd: 0.114 µg/L Hg: nd	Turkey	Saribal (2019)
	Undefined	Thyme	Pb: 0.801 mg/kg Cd: 0.138 mg/kg Cr: 1.025 mg/kg	Nigeria	Onyema et al. (2015)
		Nutmeg	Pb: 0.488 mg/kg Cd: 0.083 mg/kg Cr: 0.787 mg/kg		
		Curry powder	Pb: 0.372 mg/kg Cd: 0.062 mg/kg Cr: 0.638 mg/kg		
		Ginger	Pb: 0.435 mg/kg Cd: 0.062 mg/kg Cr: 0.905 mg/kg		
Paraben	Plastic	Olives	MP: <LOQ‐5.2 µg/kg EP: 13.6–29.2 µg/kg PP: nd‐65.5 µg/kg BP: nd‐85.2 µg/kg	Spain	Gálvez‐Ontiveros et al. (2021)
		Puffed rice cake with chocolate	MP: <LOQ EP: 37.1 µg/kg PP: 5.5 µg/kg BP: 31.2 µg/kg		
		Sausage (hot dogs)	MP: 6.8 µg/kg EP: nd PP: nd BP: nd		
		Croissants	MP: nd‐<LOQ EP: nd PP: nd‐1.5 µg/kg BP: nd‐4.3 µg/kg		
		Burger bun	MP: 10.9 µg/kg EP: 20.1 µg/kg PP: nd BP: nd		
	Can	Canned tuna in oil	MP: nd‐<LOQ EP: nd‐146.9 µg/kg PP: nd BP: nd‐25.5 µg/kg		
	Plastic + foil + paperboard	Chips (sour cream and onion)	MP: 7.7 µg/kg EP: nd PP: nd BP: nd		
	Undefined	Ketchup	MP: 388.85 µg/L EP: nd PP: nd	India	Pradhan et al. (2020)
		Jam	MP: 416.32 µg/L EP: nd PP: nd		
		Wafer	MP: 172.94 µg/L EP: 116.49 µg/L PP: nd		
		Mayonnaise	MP: 17.52 µg/L EP: 147.68 µg/L PP: nd		
		Buttermilk	MP: 524.90 µg/L EP: nd PP: nd		

Abbreviations: Al, aluminum; BBP, benzyl butyl phthalate; BP, butyl paraben; BPA; bisphenol A; Cd, cadmium; Cr, chromium; DBP, dibutyl phthalate; DEHP, di‐2‐ethylhexyl phthalate; DEP, diethyl phthalate; DiBP, diisobutyl phthalate; DMP, dimethyl phthalate; DnBP, di‐n‐butyl phthalate; EP, ethyl paraben; Hg, mercury; LOQ, limit of quantification; MP, methyl paraben; nd, not detected; Pb, lead; PP, propyl paraben.

## 5 Measures to Reduce Migration

People can be exposed to many chemicals with the food they consume on a daily basis. Although these pollutants may not be as immediately harmful as direct ingestion of toxic substances, prolonged and continuous exposure can lead to significant health issues over time. Therefore, it is crucial to implement measures to minimize potential risks and ensure long‐term safety.

With today's technology, “smart packaging” and “active packaging” methods are emerging as innovative solutions to address packaging‐related concerns. Smart packaging monitors the product throughout all stages until it reaches the consumer, providing real‐time information about its quality and safety. Active packaging, on the other hand, incorporates functional components into the packaging material or includes separate elements within the packaging to enhance its performance. Both methods contribute to extending shelf life and improving product safety (Karakoç and Dikmen 2024). However, despite these advantages, the adoption of these technologies is limited due to concerns over the potential consumption of active or smart substances by consumers and the relatively high cost of implementation (Karakoç and Dikmen 2024). In response to these limitations, it is also important to highlight the technology referred to as “non‐migratory” packaging. This functional packaging system involves the immobilization of active substances onto the surface of the packaging material, rather than allowing them to migrate into the food. Compared to conventional active packaging systems, this approach has been shown to be effective in preventing undesirable changes in food, blocking the transfer of preservatives, and prolonging the functional efficacy of active agents. As a result, the antimicrobial and antioxidant properties are maintained over time, contributing significantly to food quality and safety (Liu et al. 2024).

When comparing packaging materials, glass stands out as one of the safest options in terms of migration risk, whereas metal packaging, also considered an inert material, offers another effective choice for reducing foodborne migration. Among plastic packaging materials, certain types should be avoided due to their association with harmful chemicals like BPA. Specifically, plastics labeled as “3” or “PVC” and “7” or “PC” are known to produce BPA, making them less safe for use. On the other hand, plastics marked “1,” “2,” “4,” and “5” can be classified as safer options, as they do not release BPA. PVC, commonly used in combination with phthalates—a toxic chemical—poses additional concerns. BPA migration is particularly significant when containers are in contact with hot or liquid food or when the material has a smooth or opaque surface (Bansal and Gupta 2020). Therefore, selecting packaging materials with these factors in mind is crucial. Further, measures to reduce migration from phthalates have been highlighted by Wang et al. (2023). These include coating the surface of packaging materials with non‐migrating substances, creating network structures within the material, incorporating nanoparticles such as silica starch nanocrystals or cellulose nanocrystals, and producing more compact structures through gamma or UV irradiation of packaging films. Applying non‐migrating coatings act as barriers, limiting the interaction between the packaging material and the food. Creating network structures within the packaging material enhances its resistance to chemical migration by restricting the mobility of harmful substances. Incorporating nanoparticles, further strengthens the material's barrier properties by filling micro‐gaps and reducing permeability. Additionally, exposing packaging films to gamma or UV irradiation creates a more compact structure, increasing material density and reducing the migration of phthalates and other contaminants. These innovative approaches, either individually or in combination, provide effective solutions to significantly reduce migration risks and enhance the safety and integrity of food packaging materials. Additional strategies involve attaching plasticizers to PVC chains and using slow‐migrating, large molecular weight plasticizers or ionic liquids. These approaches can effectively minimize migration and enhance the safety of packaging materials (Wang et al. 2023).

Environmental factors are another significant element influencing migration from packaging into food. Controlling the storage environment or selecting packaging materials appropriate for the specific food type and environmental conditions can effectively reduce migration. Key methods include maintaining optimal storage temperatures, as higher temperatures can accelerate migration; choosing packaging materials compatible with the food's pH or lipophilic properties; and minimizing storage duration to limit contact time. Alak and Yavaş (2024) investigated the effect of storage temperature on phthalate migration in vacuum‐packed fish fillets. They found that both higher storage temperatures and prolonged storage durations significantly increased the migration of DEHP. Migration rates were notably higher at elevated temperatures, emphasizing the critical role of temperature in controlling phthalate contamination in packaged foods. Their findings underscore the interplay between time and temperature in accelerating or reducing phthalate migration. Similarly, Xu et al. (2010) examined the migration of phthalates from plastic containers into cooking oil and mineral water under static and dynamic storage conditions at 20°C, 40°C, and 60°C for 2 months. They found that migration rates were significantly higher in oil due to the lipophilic nature of phthalates, with DBP showing the highest migration potential. Migration increased with higher storage temperatures (20°C, 40°C, and 60°C) and longer durations. These results underline that the lipophilic nature of phthalates contributes to higher migration in oil‐based foods, whereas factors such as higher temperatures, prolonged contact time, and dynamic storage conditions further exacerbate migration. Together, these findings emphasize the importance of optimizing storage conditions and carefully selecting packaging materials to minimize migration and ensure food safety. Additionally, to ensure food safety, it is essential to adhere to migration limits established by regulations and validate compliance through appropriate analytical methods. Moreover, migration can be reduced by designing food‐contact packaging with effective barrier structures that limit interactions and prevent chemical transfer. In paper and cardboard packaging, low molecular weight components found in printing inks are particularly prone to high levels of migration. To prevent such migration, specific barrier layers made from plastic films are often applied to block the transfer of chemicals into food (Yenidoğan et al. 2023; Aurela and Söderhjelm 2007). In a recent study, Dağdelen et al. (2024) investigated the effect of coating PVC film with biodegradable PLA on chemical migration. The results showed a general reduction in chemical migration, with a particularly notable 50% decrease in the migration of DEHP, one of the most common plasticizers. These findings suggest that PLA coating may serve as a functional barrier, effectively mitigating the migration of harmful substances from packaging into food.

## 6 Conclusion and Future Outlook

The chemical transition from packaging materials to food has become an important public health problem, especially with the increase in demand for packaged food. Current evidence suggests that phthalates and bisphenol derivatives are among the most well‐known toxic substances and are associated with serious health problems such as hormonal disorders and cancer. The necessity of conducting more in‐depth research into the known and unknown risks associated with these substances—and others yet to be identified—is of critical importance for both current and future generations. Among packaging materials, plastics stand out as the primary source of these harmful chemicals due to their widespread use and functional advantages. However, plastic should not be viewed as the sole contributor to migration risks. All components used in food packaging, including those that are not in direct contact with food, may contribute to chemical migration. Therefore, materials with adequate barrier properties should be selected. In addition to packaging materials, inks, adhesives, and other auxiliary substances should also be considered, as they can contribute to chemical transfer. Specific measures such as the use of low‐migration inks, natural adhesives, limiting the printed surface area, and reducing ink density can help minimize migration. Subsequently, migration levels must be determined through testing and analysis, ensuring that they do not exceed legally established limits.

To minimize migration and its associated health risks, practical measures should be taken to minimize transit and associated health risks, such as controlling environmental factors like mechanical stress and temperature, choosing food‐compatible packaging materials, and opting for safer alternatives such as glass. Given the numerous advantages and unique structural properties of plastic, its complete removal from daily life is unrealistic. Nevertheless, the misuse of plastic, which is widely utilized by consumers, may significantly increase potential health risks. For this reason, consumer education and awareness are essential and should not be overlooked. In addition, improvements in labeling systems that guide consumers on proper usage could contribute to safer practices. In addition, innovative solutions such as biodegradable, smart, and active packaging have emerged as promising technologies to reduce the negative effects of packaging and improve food quality. However, despite this potential, research on these advanced packaging methods remains limited, and challenges remain with their applicability.

Furthermore, advancements in barrier technologies have proven to be highly effective in reducing migration by enhancing the resistance of permeable materials such as paper and cardboard. In the case of chemically hazardous materials like plastics, these technologies can help isolate the food contact layer with safer alternatives, thereby improving food safety. Looking ahead, future efforts should focus on the development and scaling of next‐generation packaging technologies that not only ensure safety and minimize chemical migration but are also cost‐effective and environmentally sustainable. Designing multifunctional materials with superior barrier properties and reduced migration potential holds great promise for advancing both food safety and packaging performance. Moreover, collaborative research involving materials scientists, food technologists, and policymakers is critical to establishing comprehensive guidelines and improving consumer safety. Additionally, raising awareness among producers and consumers about the importance of safer packaging options and compliance with legal regulations will play a key role in reducing the risks of chemical transmission. Finally, there is an urgent need for long‐term research on the health impacts of chemical switching and the efficacy of creative packaging solutions. Public health can also be further protected by creating regulatory frameworks that address new chemical hazards and guarantee worldwide compliance. The food sector may achieve a balance between public safety and functional packaging by resolving these issues and promoting multidisciplinary research, opening the door to a more sustainable and healthful future.

## Author Contributions

**Nurbanu Seref**: investigation, conceptualization, writing–original draft. **Gizem Cufaoglu**: conceptualization, investigation, writing–review and editing, supervision.

## Conflicts of Interest

The authors declare no conflicts of interest.

## Declaration of Generative AI and AI‐Assisted Technologies in the Writing Process

During the preparation of this work, the authors used ChatGPT to assist with grammar editing and improving the overall readability of the manuscript. Following its use, the authors carefully reviewed and revised the content as necessary and take full responsibility for the final version of the publication.
